# Exploring the role of gender on treatment outcomes in older adults with alcohol use disorder

**DOI:** 10.1111/acer.70164

**Published:** 2025-09-10

**Authors:** Jeppe Sig Juelsgaard Tryggedsson, Kjeld Andersen, Silke Behrendt, Michael P. Bogenschutz, Gerhard Buehringer, Anette Søgaard Nielsen

**Affiliations:** ^1^ Research Unit of Psychiatry, Department of Clinical Research University of Southern Denmark Odense Denmark; ^2^ Department of Mental Health Odense Region of Southern Denmark Odense Denmark; ^3^ OPEN, Odense Patient Data Explorative Network Odense University Hospital Odense Denmark; ^4^ Department of Psychology University of Southern Denmark Odense Denmark; ^5^ NYU Langone Medical Center New York New York USA; ^6^ Health Sciences Center University of New Mexico Albuquerque New Mexico USA; ^7^ IFT Mental Health Solutions Munich Germany; ^8^ Institute of Clinical Psychology and Psychotherapy Technische Universität Dresden Dresden Germany

**Keywords:** alcohol use disorder, gender differences, motivational enhancement therapy, older adults, treatment outcomes

## Abstract

**Background:**

Alcohol use disorder (AUD) among older adults, particularly with respect to gender differences in treatment outcomes, remains underexplored. Our objective was to explore gender differences in AUD treatment outcomes among older adults, focusing on continuous measures (e.g., drinks per day) and binary measures (e.g., abstinence) across a 1‐year period.

**Methods:**

We analyzed data from a multinational randomized controlled trial involving 693 older adults (60+) diagnosed with DSM‐5 AUD. Participants received motivational enhancement therapy and the community reinforcement approach, across sites in Denmark, Germany, and the United States. Participants were assessed at baseline and after 4, 12, 26, and 52 weeks. Multilevel mixed‐effects linear and logistic regressions were used, adjusted for sociodemographic and baseline drinking characteristics.

**Results:**

Both men and women showed significant improvements across all outcomes. At baseline, females reported 0.75 fewer drinks/day, 1.33 fewer drinks/drinking day, and 50% lower odds of low blood alcohol content (BAC) compared to males (OR = 0.50; *p* < 0.05). Gender–time interactions showed smaller reductions in females' drinks per day and drinks per drinking day (*p* < 0.05), resulting in similar drinking levels at follow‐ups. No gender differences were found at any time points for percent days abstinent and percent heavy drinking days (*p* ≥ 0.05). A significant gender–time interaction was found for percent days abstinent (*p* = 0.04), but no consistent direction was observed across time points. For abstinence and no heavy drinking, no gender differences were found (*p* ≥ 0.05). No interactions between gender and time were found for any binary outcome (*p* ≥ 0.05).

**Conclusions:**

Among older adults with DSM‐5 AUD diagnosis, treatment led to substantial and sustained improvements across genders. While women showed less favorable drinking reductions, adjusted estimates were broadly comparable. Given women's increased physiological vulnerability to alcohol, this may not imply equivalent clinical risk. Still, findings support the potential for meaningful treatment benefits regardless of gender.

## Graphical abstract

This study found that older adults (60+ years) with alcohol use disorder (AUD) experienced substantial and sustained improvements in drinking outcomes 1 year after treatment. While women started with lower alcohol consumption, they showed smaller reductions over time compared to men, resulting in similar levels at follow‐up. No consistent gender differences emerged in abstinence or heavy drinking outcomes. These findings suggest that older adults of both genders benefit comparably from AUD treatment, supporting the need for inclusive, age‐tailored interventions.

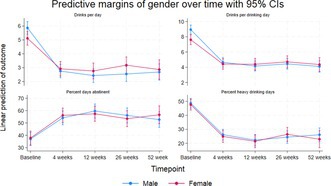

## INTRODUCTION

Alcohol use disorder (AUD) among older adults (60+ years) is a significant public health concern. Demographic changes in Western countries, with an increasing number of older adults, and given that prevalence rates of AUD already reach significant proportions in this demographic (Breslow et al., 2017; Han et al., 2017, 2019; Muñoz et al., 2018), an increase in the number of older adults with AUD is expected. Epidemiological research has observed rising trends in alcohol consumption among older adults, alongside differences between genders (Steffens et al., 2024).

Older men consume alcohol more frequently and in larger quantities compared to older women (Geels et al., 2013; Han et al., 2017, 2019). Binge drinking, which poses the highest risks for immediate and long‐term health and social issues, is also more prevalent in older men than in women (Blow, 2000; Geels et al., 2013; Han et al., 2017, 2019; Rosendahl et al., 2022). In recent years, however, an increase in alcohol consumption among older women, including instances of binge drinking, has led to a reduction in gender differences in alcohol consumption among older adults compared to what has previously been observed (Han et al., 2017, 2019; White, 2020). Furthermore, older adults experience an increased sensitivity to the intoxicating effects of alcohol intake compared to younger adults, and older women are more sensitive than older men (Erol & Karpyak, 2015). Still, prevalence estimates reveal a higher proportion of older men suffering from AUD compared to older women (Han et al., 2017).

These gender differences among older adults are expressed by higher rates of hospitalization and mortality among men (Hallgren et al., 2009). Research also indicates that older women entering treatment for AUD are more likely to have experienced a later onset of AUD than men (Dauber et al., 2018). Additionally, the experience of AUD may carry a greater burden of stigma and shame for women compared to men (Epstein et al., 2007), and generally, the consequences of AUD appear to be more negative for women than for men (Erol & Karpyak, 2015; Nolen‐Hoeksema, 2004). Several factors likely contribute to these potential gender differences in alcohol use and treatment seeking. For example, in terms of life transitions and social factors, older women are more commonly widowed, reside alone, and exhibit higher rates of retirement in comparison with older men (Dauber et al., 2018; Tryggedsson et al., 2023).

Overall, treatment for problematic alcohol use and AUD has been shown to be equally effective in older adults as it is in other age groups (Andersen et al., 2019; Dauber et al., 2018; Lemke & Moos, 2002, 2003; Oslin et al., 2002, 2005; Satre, Mertens, Areán, et al., 2004). Some studies even suggest better adherence and drinking outcomes among older adults compared to younger and middle‐aged adults (Bhatia et al., 2015; Kuerbis & Sacco, 2013; Oslin et al., 2002; Satre, Mertens, Areán, et al., 2004). However, knowledge of the influence of gender on AUD treatment outcomes is limited, and so far, the literature has shown mixed results. Older studies suggested that older women achieve better outcomes on some drinking‐related measures compared to older men, following treatment for alcohol dependence (Satre et al., 2007; Satre, Mertens, Areán, et al., 2004; Satre, Mertens, & Weisner, 2004). Yet, these study samples included only a small number of older women, resulting in limited power and making extensive gender comparisons impractical. In 2009, Adamson et al. identified 18 studies focused on the role of gender in predicting alcohol treatment outcomes. They found that eight studies reported more favorable outcomes for women, while only one study showed similar results for men. Notably, these studies did not specifically target older populations and employed a range of methodological approaches, including various treatment outcomes. More recently, results from a large‐scale clinical trial of patients with AUD aged 60 years and older indicated a higher likelihood of treatment success for male patients compared to females (Andersen et al., 2019). In the given study, treatment success was defined as abstinence or maintaining a blood alcohol content (BAC) of 0.05% or lower at any point during the 30 days leading up to a 26‐week follow‐up period—an outcome measure not evaluated in any of the studies reviewed by Adamson et al. (2009). This finding further complicates the understanding of gender differences in treatment outcomes, as it conflicts with earlier results within this age group (Satre et al., 2007; Satre, Mertens, Areán, et al., 2004; Satre, Mertens, & Weisner, 2004). Therefore, there is a compelling need to reanalyze data from the study by Andersen et al. (2019) to broaden the investigation beyond the single binary outcome of BAC. Our study intends to examine a spectrum of alcohol‐related outcomes—both continuous and binary measures—as recommended by Witkiewitz et al. (2015). Examining whether the gender differences observed by Andersen et al. persist across a wider set of outcome measures could help identify underlying mechanisms driving these differences. By addressing the current lack of literature on gender‐specific treatment responses in older adults, our study contributes to improving treatment strategies for this population, potentially leading to more effective interventions.

The overall aim of this study was to investigate the role of gender on a variety of AUD treatment outcomes among older adults aged 60 years and above through a 1‐year follow‐up period. The specific objectives were (1) to assess the association between gender and continuous drinking‐related outcome measures, specifically drinks per day, drinks per drinking day, the percentage of days abstinent, and the percentage of heavy drinking days; and (2) to examine the relationship between gender and binary drinking‐related outcome measures, specifically abstinence, no heavy drinking, and a low BAC.

## MATERIALS AND METHODS

### Study design and setting

This study utilized AUD treatment data from the Elderly Study; a multinational, multicenter, randomized, controlled, single‐blind clinical trial, evaluating the intervention of adding a community reinforcement approach for seniors (CRA‐S) to brief outpatient motivational enhancement therapy (MET) treatment for older adults with DSM‐5 AUD (ClinicalTrials.gov ID: NCT02084173). The design, setting, participant selection, and primary results of the trial have been described in detail elsewhere (Andersen et al., 2015, 2019; Søgaard Nielsen et al., 2016).

In summary, the trial was carried out in three countries from January 2014 to May 2016: Denmark (Copenhagen, Aarhus, and Odense), Germany (Munich and Dresden), and the United States (Albuquerque, New Mexico). Patients aged 60 years or older who met the DSM‐5 criteria for AUD (American Psychiatric Association, 2013) were invited to participate in the trial. These patients were identified through various pathways: self‐referral, referrals from general practice or hospitals in Denmark and Germany, or from positive alcohol problem screenings during clinical evaluations at a primary care site in the United States. All AUD treatment was provided free of charge. The trial's exclusion criteria were (a) the inability to give at least 8 of 10 correct answers in an informed consent comprehension quiz, and therefore indicating failure to fully understand the implications of participation or even cognitive impairment, (b) psychotic symptoms at the time of inclusion, (c) severe depression at the time of inclusion, (d) a lifetime diagnosis of bipolar disorder, (e) suicidal thoughts or behavior at the time of inclusion, (f) the use of illegal opioids or stimulants (however, other types of medication were allowed, e.g., medically prescribed opioids), (g) participation in other alcohol treatment within 30 days prior to the time of inclusion; however, this did not apply to treatments with the sole purpose of detoxification, and (h) patients with legally authorized representatives.

#### Interventions

Patients were randomized to receive either MET (*n* = 351) or MET + CRA‐S (*n* = 342). Thus, all participants (*N* = 693) received brief, manualized, outpatient AUD treatment based on the MET approach—a technique often used in the treatment of alcohol and substance use disorders (Arkowitz et al., 2015). During the planning phase of the Elderly Study, the development and refinement of the intervention's content was supported by US‐based MET specialists, ensuring fidelity to established principles and alignment with international best practices (Andersen et al., 2015). The MET approach integrates motivational interviewing (MI), feedback on assessment results, and strategies from cognitive behavioral therapy (CBT) to help develop motivation to change drinking behavior, including enhanced self‐efficacy (Hettema et al., 2005; Miller & Rollnick, 2012). In the Elderly Study, MET treatment consisted of four sessions, about once each week, each lasting between 60 and 90 min, over a total duration of about 4 weeks. The content of the four MET sessions consisted of an exploration of the patient's alcohol use, feedback, a brief functional analysis of alcohol use, development of a personal change plan, and, if possible, involvement of a significant other in the fourth and final session. MET + CRA‐S treatment consisted of up to eight additional treatment sessions following the initial four MET sessions described above. These subsequent sessions utilized a manualized community reinforcement approach (Miller et al., 1999) age‐adapted for seniors (CRA‐S). Each session lasted approximately 60 min and included one or more modules aimed at developing skills and strategies to support a positive, rewarding lifestyle free from alcohol use. The community reinforcement approach (CRA) also makes use of several procedures well‐known from CBT (Meyers et al., 2011). More specifically, the CRA approach focuses on identifying rewarding and prosocial behaviors, and on teaching patients' skills and strategies to help them build a fulfilling lifestyle without problematic alcohol use (Khalid et al., 2024; Morse et al., 2020). In the Elderly Study, CRA‐S was delivered as a flexible set of modules that patients could integrate into their treatment plan, which was developed during the final MET session. Examples of CRA‐S modules included coping with cravings, building a sober network, and engaging in social activities. For the Elderly Study, a specialized CRA‐S module focusing on coping with aging was developed and could be included in the eight additional CRA‐S sessions, based on patient preference (Andersen et al., 2015). This module addressed challenges associated with aging, recognizing older age as a period of transitions often accompanied by losses—such as retirement, bereavement, and reduced social networks—which may increase the risk of loneliness, defined as the emotional response to isolation or lack of companionship. Two coping strategies were introduced: problem‐focused and emotion‐focused coping. The former involved identifying and analyzing specific problems, exploring alternative solutions, and taking active steps to change the situation. The latter aimed to help patients manage emotional distress by consciously regulating emotions, redirecting thoughts, and engaging in relaxation techniques or pleasant activities to reduce stress and promote psychological resilience. All sessions in the Elderly Study were audio‐recorded.

The therapists were either experienced clinicians from local treatment centers—typically social workers, psychologists, or psychiatric nurses with additional training in CBT and MI—or master's‐level psychologists in training to become state‐licensed clinical psychologists. All therapists were trained in both treatment conditions and completed intensive training prior to study initiation. Ongoing supervision was provided based on randomly selected session recordings, and regular video meetings among supervisors ensured consistency in supervision across study sites. In total, 47 therapists participated in the study.

Fidelity ratings in the Elderly Study, which assessed the adherence of therapists to the principles and techniques of MI, were high and met established benchmarks for effective MI (Schmidt et al., 2019), indicating that treatment was delivered both correctly and consistently. All sessions and modules were thoroughly described in manuals for the Elderly Study, available online: MET treatment alone (Moyers et al., 2013b) and MET + CRA‐S treatment (Moyers et al., 2013a).

### Study sample

For this study, we included all participants in our study sample. As participants were followed up at multiple time points, we included the maximum number of participants from each follow‐up. Thus, relative to the baseline sample of 693 participants, response rates were 638 (92.1%) after 4 weeks, 602 (86.9%) after 12 weeks, 544 (78.5%) after 26 weeks, and 508 (73.3%) after 52 weeks. All missing values were handled by means of multiple imputation. Thus, full information was available at all time points.

#### Baseline and follow‐up assessments

Information on sociodemographic factors was collected through questionnaires at baseline, and information on alcohol consumption measures was collected at baseline and multiple follow‐ups. Follow‐ups were conducted after 4 weeks, where MET treatment was completed, after 12 weeks, where MET + CRA‐S treatment was completed, after 26 weeks, and after 52 weeks. Follow‐up assessments were scheduled based on the start of each participant's baseline, regardless of the treatment length.

Information on sociodemographic factors included participants' age, country, level of education, employment, and marital status. Furthermore, information on previous treatment episodes and level of alcohol dependency according to the Alcohol Dependency Scale (ADS; Skinner & Allen, 1982) was collected. Alcohol consumption was measured through the Form 90‐AI/F instrument (Miller & Del Boca, 1994), at baseline and all follow‐ups.

#### Measures of sociodemography and dependency

Information on sociodemographic factors was collected using a customized questionnaire. Participants' age was used as a continuous variable. Level of education was categorized as *No degree*, *At most undergraduate*, or *Graduate/postgraduate*. Employment status was categorized as *Working* (participants reporting full time or part time work) or *Not working* (participants reporting homemaker, retirement, unemployment, or disability). Marital status was categorized as being *Single* (participants reporting being single and never married, separated but still married, divorced, or widowed) or *Cohabiting* (participants reporting being married or living with partner but not married). To assess whether participants had a previous treatment episode, they were asked “Have you ever been in treatment (inpatient or outpatient) for addiction problems?” The variable was then categorized as *No* or *Yes* (for participants reporting one or more episodes). Lastly, level of alcohol dependency was categorized as *None or mild dependency* (0–13), *Moderate dependency* (14–21), or *Substantial/severe dependency* (22 or higher).

#### Outcome measures

Alcohol consumption was quantified as the number of daily standard drinks (equivalent to 12 grams of pure alcohol) over the 30 days preceding each assessment (baseline and follow‐up). This information was used to calculate drinking variables as described below. The selection of outcome measures for this study was based on recommendations for the design and analysis of treatment trials for AUD made by Witkiewitz et al. (2015). The addition of the binary outcome measure low BAC (BAC ≤0.05% during the last 30 days) was based on it being the primary outcome measure at the 26‐week follow‐up in the Elderly study.

##### Continuous outcomes

- Drinks per day: Mean number of standard drinks per day within the last 30 days prior to assessment.
- Drinks per drinking day: Mean number of standard drinks per drinking day within the last 30 days.
- Percent days abstinent: Percentage of days with no alcohol consumption within the last 30 days.
- Percent heavy drinking days: Percentage of heavy drinking days within the last 30 days, defined as 4+ standard drinks for women and 5+ standard drinks for men.

##### Binary outcomes

- Abstinence: Indicating whether participants reported any alcohol consumption in the 30 days prior to assessment. Categorized as *Yes* (no consumption) or *No* (any consumption).
- Any heavy drinking: Based on whether participants reported any heavy drinking days within the last 30 days. Categorized as *Heavy drinking* or *No heavy drinking*.
- Low BAC: A blood alcohol content (BAC) ≤0.05% was categorized as *Yes* for participants whose BAC did not exceed 0.05% in the 30 days prior to assessment or *No* for those where it did. BAC was calculated using the Widmark formula (Posey & Mozayani, 2007); thus, based on the total amount and duration of alcohol consumption, a gender‐specific constant, and weight.

### Statistical analyses

Pearson's chi‐squared tests were used to analyze categorical baseline covariates across genders. Continuous baseline covariates were compared between genders using the Wilcoxon rank‐sum test (Mann–Whitney). This approach was taken since none of the continuous variables were normally distributed. To describe sample characteristics in Table 1, we reported the median and the interquartile range.

**TABLE 1:** Baseline characteristics of the study sample stratified by gender.

	Gender		Total	Test^a^
	Male	Female		
Participants, *n* (%)	414 (59.7%)	279 (40.3%)	693 (100.0%)	
Age in years, median (IQR)	64 (6)	65 (7)	64 (6)	0.038
Country, *n* (%)				
Danish Site	219 (52.9%)	122 (43.7%)	341 (49.2%)	0.022
New Mexico Site	89 (21.5%)	60 (21.5%)	149 (21.5%)	
German Site	106 (25.6%)	97 (34.8%)	203 (29.3%)	
Education, *n* (%)				
No degree	94 (22.7%)	72 (25.8%)	166 (24.0%)	0.035
At most undergraduate	211 (51.0%)	157 (56.3%)	368 (53.1%)	
Graduate/postgraduate	109 (26.3%)	50 (17.9%)	159 (22.9%)	
Employment status, *n* (%)				
Working	86 (20.8%)	56 (20.1%)	142 (20.5%)	0.823
Not working	328 (79.2%)	223 (79.9%)	551 (79.5%)	
Marital status, *n* (%)				
Single	193 (46.6%)	175 (62.7%)	368 (53.1%)	<0.001
Cohabiting	221 (53.4%)	104 (37.3%)	325 (46.9%)	
Previous treatment episodes for AUD, *n* (%)				
No	221 (53.4%)	168 (60.2%)	389 (56.1%)	0.075
Yes	193 (46.6%)	111 (39.8%)	304 (43.9%)	
Alcohol dependence, *n* (%)				
None or mild	301 (72.9%)	196 (70.8%)	497 (72.0%)	0.440
Moderate	93 (22.5%)	62 (22.4%)	155 (22.5%)	
Severe	19 (4.6%)	19 (6.9%)	38 (5.5%)	
Drinks per day, median (IQR)	5.1 (6.4)	3.7 (4.5)	4.3 (5.7)	<0.001
Treatment group, *n* (%)				
MET	213 (51.4%)	138 (49.5%)	351 (50.6%)	0.608
MET + CRA‐S	201 (48.6%)	141 (50.5%)	342 (49.4%)	
Drinks per drinking day, median (IQR)	7.8 (7.7)	5.8 (5.2)	6.9 (6.7)	<0.001
Percent days abstinent, median (IQR)	30.0 (70.0)	36.7 (73.3)	30.0 (70.0)	0.201
Percent heavy drinking days, median (IQR)	46.8 (80.0)	43.3 (73.3)	46.7 (76.7)	0.278
Abstinence, *n* (%)				
No	377 (91.1%)	253 (90.7%)	630 (90.9%)	0.864
Yes	37 (8.9%)	26 (9.3%)	63 (9.1%)	
Any heavy drinking, *n* (%)				
Heavy drinking	346 (83.6%)	232 (83.2%)	578 (83.4%)	0.884
No heavy drinking	68 (16.4%)	47 (16.8%)	115 (16.6%)	
Low BAC, *n* (%)				
No	298 (72.0%)	233 (83.5%)	531 (76.6%)	<0.001
Yes	116 (28.0%)	46 (16.5%)	162 (23.4%)	

*Note*: A standard drink = 12 g of pure alcohol. Heavy drinking was defined as four or more drinks/day for females and five or more for males.

Abbreviation: IQR, interquartile range.

^a^ For continuous variables, comparisons between males and females were performed using the Wilcoxon rank‐sum test (Mann–Whitney). For categorical variables, the chi‐squared test was used.

To investigate the impact of gender on continuous treatment outcomes during and after treatment, we employed multilevel mixed‐effects linear regressions. These analyses were conducted both as crude (including gender, time, a gender‐time interaction term, and the baseline outcome value) and as fully adjusted, additionally considering age, level of education, employment status, marital status, history of previous treatment, level of alcohol dependency, and treatment group. Despite the Elderly Study's findings indicating no significant differences between intervention groups at the 6‐month follow‐up, treatment group was retained as a covariate to control for any potential confounding effects. This included variations in treatment length by study condition, as treatment attendance was otherwise not accounted for in the analyses. Our models primarily focused on the gender‐related change in treatment outcomes, comparing females to males, rather than the absolute differences between genders at certain assessment time points. This approach emphasized the influence of gender on the development of treatment outcomes. To investigate gender differences in the change of outcome measures from baseline to follow‐ups, an interaction term between gender and time was included in both the crude and adjusted models. The significance of the interaction was tested using likelihood‐ratio tests. For binary treatment outcomes, we analyzed the gender effects using multilevel mixed‐effects logistic regressions—both in crude and adjusted formats and with the same approach as described above. Using multilevel mixed‐effects regression models allowed us to utilize data from all time points. All models included both random intercepts and slopes to account for individual variability in baseline treatment outcomes and changes over time points.

Regarding missing data on alcohol consumption measures, we addressed this through multiple imputation by chained equations (MICE), utilizing linear, logistic, and ordered logistic regression methods for imputation (Bartlett et al., 2015; Rubin, 1987). The imputation model included age, gender, educational level, employment status, marital status, previous treatment episodes, and treatment group as auxiliary variables, generating a total of 10 imputed datasets for analysis. Although alcohol consumption measures might not necessarily be missing at random (MAR), this approach was considered meaningful, as previous studies have demonstrated that multiple imputation is a feasible method to deal with missing data in clinical trials of treatments for AUD, including attrition and nonresponse for both continuous and binary outcomes (Hallgren et al., 2016; Hallgren & Witkiewitz, 2013; Witkiewitz et al., 2014). In fact, multiple imputation is considered one of the better and more superior methods to handle missing data (Harris et al., 2013). To evaluate the robustness of our imputation strategy, we reran the analyses on the nonimputed data and compared these results with the imputed results. We also performed attrition analyses to see whether gender predicted missingness. Neither of the analyses yielded any significant results and are therefore not presented.

All statistical analyses were performed using STATA version 18.0, and a significance level was set at *p* = 0.05.

## RESULTS

### Sample characteristics

Table 1 provides a comprehensive overview of the baseline characteristics of the study sample, emphasizing significant sociodemographic and baseline consumption differences between genders. The sample included *N* = 693 older adults diagnosed with AUD, comprising 59.7% male and 40.3% female participants. The median age was 64 years (interquartile range [IQR] = 6), with female participants being slightly older (65 years, IQR = 7.0, range [60–81]) than male participants (64 years, IQR = 6.0, range: 60–86, *p* = 0.04). Most of the participants were from Denmark, with 341 individuals, representing 49.2% of the total study sample. The German sites contributed 203 participants, making up 29.3% of the total, while the United States had the smallest cohort, with 149 participants, accounting for 21.5% of the total. At the Danish sites, a smaller proportion of women were enrolled compared to men; only 35.8% of participants were female. This contrasts with the German sites, where the largest proportion of women was observed, with females comprising 47.8% of the participants. Significant baseline differences between genders were also observed in other sociodemographic factors such as level of education, where a larger proportion reported higher education among men compared to women (*p* = 0.04), and marital status, where a larger proportion reported being single in females compared to males (*p* < 0.01). There was some evidence of a larger proportion of men previously having received AUD treatment compared to women; however, this result was not statistically significant (*p* = 0.08).

Mean values for continuous drinking outcomes by gender and time points after multiple imputation are provided as Table S1. At baseline, males reported consuming more drinks per day on average compared to females, with median values of 5.1 for males and 4.3 for females (*p* < 0.01). Similarly, males reported a higher average number of drinks per drinking day than females, with medians of 7.8 for males and 6.9 for females (*p* < 0.01). In terms of the percentage of days abstinent, no significant differences were observed between genders (total median = 30%, *p* = 0.20). We also did not observe any significant differences in the percentage of heavy drinking days between males and females (total median = 46.7%, *p* = 0.28). The proportion of participants reporting abstinence during the 30 days prior to treatment start was similar for both genders, with a total of 9.1% (*p* = 0.86). Proportions reporting no heavy drinking were also comparable between genders, at a total of 16.6% (*p* = 0.88). Lastly, a significantly higher percentage of males (28.0%) reported a low BAC compared to females (16.5%, *p* < 0.01).

### Treatment outcomes

In addition to the text below, Tables 2 and 3 provide detailed summaries of the crude and adjusted effects of gender, time, and the gender–time interaction on the continuous and binary treatment outcomes, respectively. To assess the robustness of our findings, we reanalyzed all models using nonimputed data (complete case analyses). Results were consistent with those based on imputed datasets, with no substantial differences in estimates or confidence intervals. Attrition analyses showed no significant association between gender and missingness at any time point. Full results from these supplementary analyses are available upon request.

**TABLE 2:** Crude and adjusted multilevel mixed‐effects linear regression coefficients and 95% confidence intervals for gender, time, and gender–time interaction of continuous outcomes over time points (up to 52‐weeks).

	Drinks per day						Drinks per drinking day						Percent days abstinent						Percent heavy drinking days					
	Crude			Adjusted			Crude			Adjusted			Crude			Adjusted			Crude			Adjusted		
	*β*	95% CI		*β*	95% CI		*β*	95% CI		*β*	95% CI		*β*	95% CI		*β*	95% CI		*β*	95% CI		*β*	95% CI	
Gender																								
Female	−0.79	−1.19	−0.40	−0.75	−1.16	−0.34	−1.30	−1.88	−0.73	−1.33	−1.91	−0.74	1.25	−2.15	4.65	0.82	−2.65	4.28	−1.34	−4.85	2.16	−1.02	−4.59	2.55
Time																								
4 weeks	−3.12	−3.47	−2.76	−3.12	−3.49	−2.76	−4.29	−4.80	−3.78	−4.31	−4.82	−3.79	17.12	14.00	20.25	17.14	14.00	20.28	−22.52	−25.75	−19.29	−22.60	−25.87	−19.32
12 weeks	−3.44	−3.81	−3.06	−3.43	−3.80	−3.06	−4.77	−5.32	−4.22	−4.73	−5.27	−4.20	22.69	19.42	25.97	22.44	19.15	25.72	−26.48	−29.82	−23.14	−26.44	−29.78	−23.09
26 weeks	−3.32	−3.74	−2.91	−3.39	−3.80	−2.98	−4.49	−5.06	−3.92	−4.61	−5.17	−4.05	19.14	15.44	22.84	19.65	15.94	23.36	−24.28	−28.01	−20.55	−24.66	−28.36	−20.95
52 weeks	−3.18	−3.65	−2.71	−3.20	−3.66	−2.73	−4.83	−5.47	−4.19	−4.84	−5.47	−4.20	15.74	11.64	19.84	15.91	11.78	20.05	−22.61	−26.91	−18.31	−22.81	−27.06	−18.55
Gender # Time																								
Female # 4 weeks	0.92	0.36	1.48	0.92	0.36	1.49	1.10	0.29	1.91	1.09	0.27	1.90	1.22	−3.66	6.10	1.31	−3.55	6.17	−0.28	−5.40	4.83	−0.20	−5.39	4.99
Female # 12 weeks	1.08	0.48	1.69	1.09	0.49	1.70	1.55	0.62	2.49	1.54	0.65	2.43	−3.08	−8.30	2.14	−2.91	−8.11	2.28	0.23	−5.13	5.58	0.39	−4.93	5.71
Female # 26 weeks	1.39	0.73	2.04	1.43	0.77	2.08	1.59	0.64	2.54	1.56	0.63	2.49	−3.47	−9.24	2.31	−3.23	−9.05	2.60	3.01	−3.02	9.05	3.22	−2.89	9.32
Female # 52 weeks	0.93	0.20	1.67	0.95	0.22	1.68	1.58	0.57	2.58	1.56	0.58	2.55	3.09	−3.28	9.46	3.09	−3.42	9.59	−2.15	−8.78	4.48	−1.85	−8.42	4.72
*p*‐Value, interaction term	<0.001			<0.001			0.005			0.005			0.040			0.041			0.4877			0.4882		

*Note*: Crude models included gender, time, gender–time interaction term, and the baseline value of the outcome measure. Adjusted models additionally included age, educational, employment, marital status, previous treatment, alcohol dependency level, and treatment group. Significance of the interaction term was tested using likelihood‐ratio tests. A *p*‐value of <0.05 was considered statistically significant. Reference groups: Gender, Male; Time, Baseline; and Gender # Time, Female # Baseline.

Abbreviations: CI, confidence interval; *β*, regression coefficient.

**TABLE 3:** Crude and adjusted multilevel mixed‐effects logistic regression coefficients and 95% confidence intervals for gender, time, and gender–time interaction of binary outcomes over time points (up to 52‐weeks).

	Abstinence						No heavy drinking						Low BAC					
	Crude			Adjusted			Crude			Adjusted			Crude			Adjusted		
	OR	95% CI		OR	95% CI		OR	95% CI		OR	95% CI		OR	95% CI		OR	95% CI	
Gender																		
Female	0.95	0.45	2.01	0.88	0.42	1.85	1.02	0.59	1.79	0.97	0.56	1.70	0.52	0.31	0.86	0.50	0.30	0.83
Time																		
4 weeks	6.16	3.67	10.36	6.14	3.66	10.31	8.00	5.29	12.12	8.00	5.28	12.11	4.33	3.00	6.27	4.34	3.00	6.28
12 weeks	9.23	5.49	15.54	9.21	5.48	15.49	10.56	6.90	16.18	10.56	6.89	16.16	6.39	4.36	9.37	6.40	4.37	9.39
26 weeks	5.82	3.43	9.86	5.80	3.42	9.82	8.98	5.90	13.67	8.97	5.90	13.65	5.88	3.97	8.72	5.89	3.97	8.73
52 weeks	5.37	3.17	9.10	5.35	3.16	9.06	7.59	4.94	11.68	7.59	4.93	11.66	7.56	5.08	11.27	7.58	5.08	11.30
Gender # Time																		
Female # 4 weeks	1.42	0.61	3.27	1.39	0.61	3.20	1.29	0.67	2.48	1.28	0.66	2.46	1.83	1.00	3.35	1.81	0.99	3.32
Female # 12 weeks	0.86	0.38	1.97	0.85	0.37	1.93	0.93	0.48	1.83	0.93	0.48	1.81	0.99	0.52	1.86	0.98	0.52	1.84
Female # 26 weeks	1.22	0.52	2.85	1.20	0.51	2.79	0.84	0.44	1.62	0.84	0.44	1.61	1.16	0.59	2.28	1.15	0.59	2.27
Female # 52 weeks	1.31	0.57	3.03	1.29	0.56	2.97	1.47	0.75	2.89	1.46	0.74	2.87	1.53	0.79	2.94	1.51	0.78	2.92
*p*‐Value, interaction term	0.605			0.610			0.296			0.297			0.157			0.161		

*Note*: Crude models included gender, time, gender–time interaction term, and the baseline value of the outcome measure. Adjusted models additionally included age, educational, employment, marital status, previous treatment, alcohol dependency level, and treatment group. Significance of the interaction term was tested using likelihood‐ratio tests. A *p*‐value of <0.05 was considered statistically significant. Reference groups: Gender, Male; Time, Baseline; and Gender # Time, Female # Baseline.

Abbreviations: CI, confidence interval; OR, odds ratio.

#### Continuous measures

**FIGURE 1:**
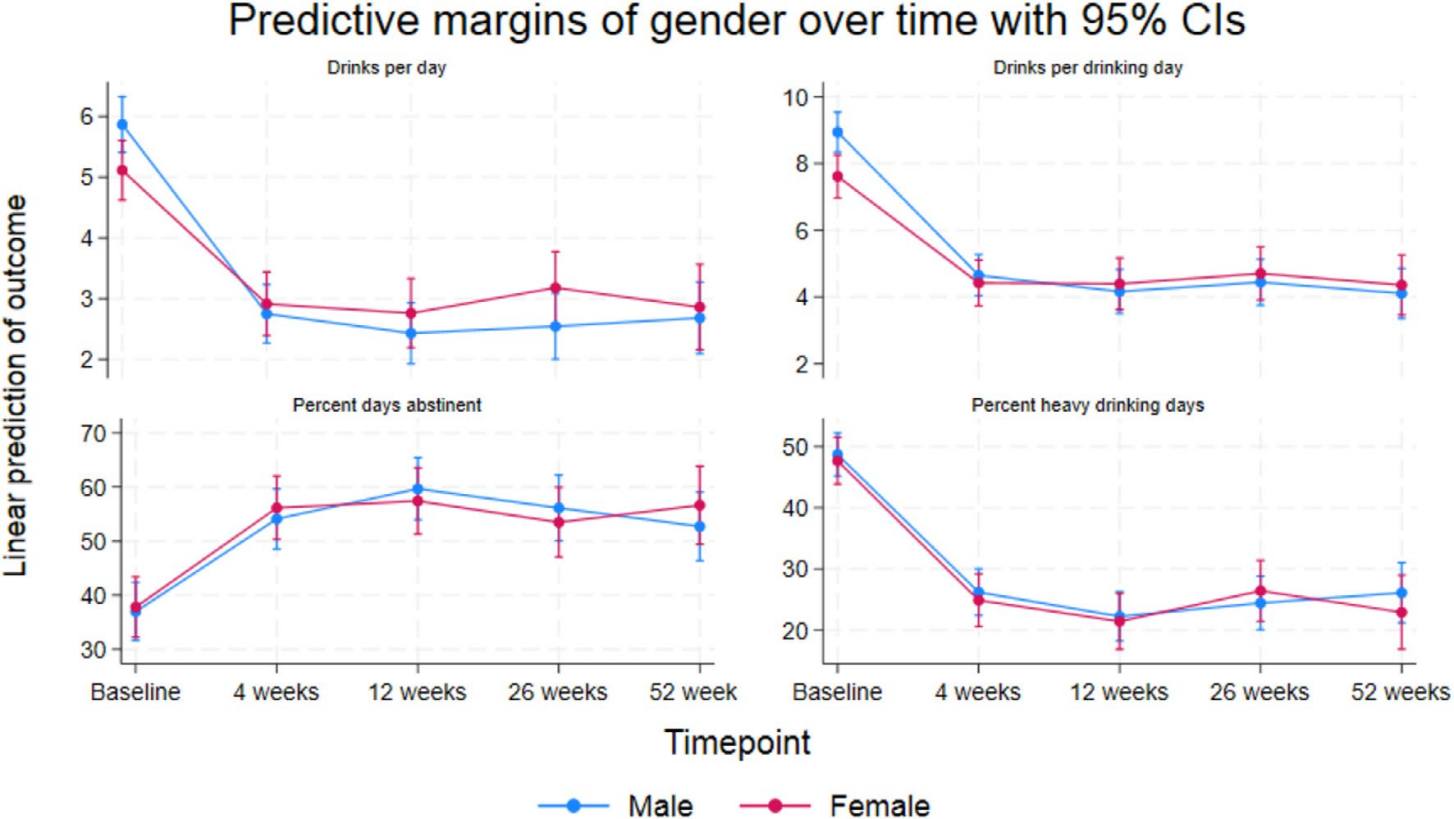
Adjusted trajectories of continuous alcohol use disorder (AUD) treatment outcomes over a 1‐year follow‐up period, stratified by gender.

In examining the effect of gender on drinks per day and drinks per drinking day, the crude and adjusted analyses showed consistent results. There was a consistent and significant reduction in drinks per day across time (all *p* < 0.01), suggesting an overall improvement or reduction in the number of drinks per day (Figure 1). The adjusted analysis revealed a statistically significant baseline difference of −0.75 drinks per day for females compared to males (*p* < 0.01). However, the interaction term indicated that gender plays a significant role in how the number of drinks per day changed over time points (*p* < 0.01), with females showing different and less favorable improvements at follow‐ups relative to baseline compared to males (adj. gender‐time *β*‐coeff. ranged from 0.92 at 4 weeks to 1.43 at 26 weeks), detailed in Table 2. For drinks per drinking day, we also found a significant and consistent reduction from baseline to follow‐ups (all *p* < 0.01). The adjusted analysis indicated a statistically significant baseline difference of −1.33 drinks per drinking day for females compared to males (*p* < 0.01). Again, an interaction between gender and time was observed (*p* < 0.01), with females showing less favorable changes in their number of drinks per drinking day relative to baseline compared to males (adj. gender‐time *β*‐coeff. ranged from 1.09 at 4 weeks to 1.56 at 26 and 52 weeks; see Table 2 and Figure 1). Both crude and adjusted analyses indicated similar patterns of a statistically significant and consistent increase in percent days abstinent of about 16%–22% from baseline to follow‐ups (all *p* < 0.01), as detailed in Table 2. For this outcome, we did not observe any significant baseline difference between males and females (adj. *p* = 0.64). We did find evidence of a statistically significant interaction between gender and time (*p* = 0.04); however, no consistent direction of differences could be observed across the entire follow‐up period (Table 2 and Figure 1). We found a significant and consistent reduction in percent heavy drinking days of about 23%–26% across all follow‐ups (*p* < 0.01), with the crude and adjusted models providing similar estimates (Table 2). At baseline, no significant effect of gender was observed in the percentage of heavy drinking days (adj. *p* = 0.58), and the interaction between gender and time was also nonsignificant (adj. *p* = 0.49), indicating that the proportion of heavy drinking days remained comparable between males and females throughout the study period. Figure 1 graphically compiles the results from the adjusted analyses of continuous outcome measures, providing a visual representation of the observed trends and highlighting the temporal changes and differential impacts of gender throughout the 1‐year follow‐up period.

#### Binary measures

**FIGURE 2:**
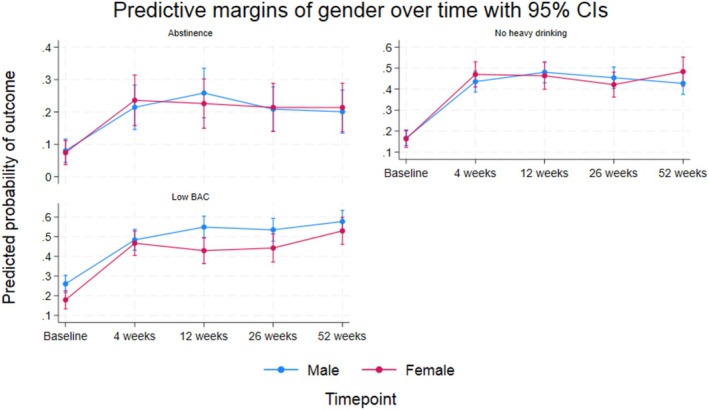
Adjusted probabilities of achieving binary alcohol use disorder (AUD) treatment outcomes over a 1‐year follow‐up period, stratified by gender.

Both crude and adjusted analyses for abstinence showed statistically significant and consistent improvements in this treatment outcome from baseline to follow‐ups, with adjusted odds ratios (ORs) ranging from 9.21 at 12 weeks to 5.35 at 52 weeks (*p* < 0.01). The analyses did not reveal any significant difference between genders at baseline (adj. OR = 0.88, *p* = 0.73). The interaction effect between gender and time was also nonsignificant (*p* = 0.61), indicating that changes in abstinence from baseline to follow‐ups were similar for both males and females (Table 3). For the outcome no heavy drinking, an improvement was observed from baseline to follow‐ups, with adjusted ORs ranging from 10.56 at 12 weeks to 7.59 at 52 weeks (*p* < 0.01). Neither the crude nor adjusted analysis showed significant baseline gender differences (adj. OR = 0.97, *p* = 0.92). The interaction between gender and time was also nonsignificant (*p* = 0.30), suggesting that both genders experienced similar tendencies in avoiding heavy drinking over the follow‐up period, as illustrated in Figure 2. Statistically significant and consistent improvements in low BAC were seen at all follow‐ups relative to baseline (all *p* < 0.01). Adjusted ORs ranged from 4.34 at 4 weeks to 7.58 at 52 weeks (Table 3). In contrast to the two other binary outcomes, a significant baseline gender difference was observed, with the adjusted analysis showing that females were significantly less likely than males to have a low BAC at baseline (OR = 0.50, *p* < 0.01). As the interaction between gender and time was not statistically significant (*p* = 0.16), the model suggested that males consistently outperformed females in maintaining a low BAC at subsequent follow‐ups. Results from the adjusted analyses of binary outcome measures are visualized in Figure 2, which complements the data presented in Table 3. Figure 2 presents adjusted probabilities of the binary outcomes, as probabilities are often easier to interpret compared to ORs.

## DISCUSSION

This study investigated the impact of gender on various continuous and binary treatment outcomes over a 1‐year follow‐up period among older adults with DSM‐5 AUD. While our results showed significant and consistent improvements across all treatment outcomes, some gender differences were present. Females reported lower intake in terms of drinks per day and drinks per drinking day at baseline compared to males. However, interaction effects indicated that females showed less favorable improvements, with smaller reductions from baseline to follow‐ups. As a result, model‐estimated values of drinks per day and drinks per drinking day became comparable between males and females at follow‐up assessments. For percent days abstinent, no significant differences were found between males and females, but the interaction between gender and time indicated some variability in abstinence patterns across time. Conversely, the percentage of heavy drinking days showed consistent improvement across time for both genders, with no significant differences observed. This suggests that treatment was not only effective in reducing heavy drinking days, but the reduction was also comparable for both males and females. The analysis of binary outcomes also yielded interesting findings. Improvements were noted in both abstinence and the avoidance of heavy drinking across time, with no significant gender differences or interaction effects, once adjusted for other factors, indicating similar trends in these outcomes between males and females. This suggests that the ability to abstain entirely from drinking and avoid heavy drinking may be similarly achievable for both genders. Conversely, a significant baseline gender difference was identified for low BAC, where females were less likely than males to have a BAC within the desired threshold of ≤0.05% at baseline. While interaction effects were not statistically significant, results could indicate that males consistently outperformed females in maintaining a low BAC throughout the 52‐week study period. While we observed statistically significant gender‐by‐time interactions for several continuous outcomes—indicating that females had smaller relative reductions over time, likely due to their lower baseline drinking levels—model‐estimated drinking values were largely similar between men and women across most outcomes by the 52‐week follow‐up. It is important to interpret these findings in light of gender‐specific risk profiles. Although most adjusted estimates of drinking behavior appeared similar at follow‐ups, women's smaller reductions and their greater physiological vulnerability to alcohol suggest that they may remain at higher risk of alcohol‐related harm, even when consuming similar amounts as men. This nuance is critical: statistical comparability in modeled drinking outcomes does not imply equivalent clinical risk (Erol & Karpyak, 2015; Nolen‐Hoeksema, 2004; Thomasson, 2002). These findings highlight the importance of understanding baseline differences and trajectories of change in tandem, while also considering both behavioral change and biological sensitivity when evaluating treatment effects across genders. Overall, our results suggest that both genders showed improvements relative to baseline, and that adjusted estimates of drinking behavior were broadly comparable between men and women across the follow‐up period.

The findings regarding low BAC should be carefully interpreted, as this measure revealed gender differences that were not observed in the outcomes “abstinence” and “no heavy drinking.” Unlike these other binary outcomes, where no significant gender differences were found, the BAC measure suggests that males are more successful than females in maintaining a BAC within the desired threshold. This discrepancy highlights the complexity of BAC as an outcome measure, which may be influenced by physiological differences in alcohol metabolism between genders, as well as other factors not captured by simpler binary outcomes. Thus, BAC can act as a nuanced indicator, especially useful in populations where abstinence and heavy drinking may not fully reflect treatment effects. Given that alcohol consumption is physiologically and metabolically different in men and women, gender differences in measured outcomes may reflect not only behavioral change but also biological variation. Clinicians and researchers should consider these nuances when using BAC as a primary indicator of treatment success, recognizing that it may reflect different aspects of alcohol use and recovery compared to measures such as abstinence and no heavy drinking. The thresholds used to define heavy drinking (≥4 standard drinks for women and ≥5 for men) align with the criteria applied in the parent trial. Alternative and more conservative definitions exist—particularly for older adults (Chen et al., 2016, National Institute on Alcohol Abuse, Alcoholism (US) & CSR Incorporated, 1991). However, to ensure consistency with the original study design and maintain comparability with prior publications, we retained the original thresholds in this secondary analysis. Although the Form 90 instrument provides detailed data covering the entire follow‐up period, we focused our analyses on the 30 days preceding each assessment. This decision was based on the original analysis plan and reflects a common approach in alcohol treatment research (Project MATCH Research Group, 1997; Sobell & Sobell, 1992; Witkiewitz & Marlatt, 2004). While longer assessment windows may offer additional insights, they were beyond the scope of the present study. Overall, our results suggest that while both males and females benefit from treatment, the pathways and extent of their recovery may differ. Treatment may differentially impact drinking behaviors between genders for certain outcomes, a relationship potentially influenced by a complex interplay of biological, psychological, and social factors (Erol & Karpyak, 2015). Nevertheless, the overall pattern of improvement observed in both men and women suggests that treatment was broadly effective across genders.

Our findings are particularly relevant in light of research indicating significant gender‐specific responses to AUD treatment in younger cohorts (Adamson et al., 2009), suggesting a potential shift in the influence of gender on treatment outcomes with age. While some older studies focusing on older adults (Satre et al., 2007; Satre, Mertens, Areán, et al., 2004; Satre, Mertens, & Weisner, 2004) have suggested better treatment outcomes for females, the existing literature on gender in alcohol treatment outcomes in general remains sparse, especially concerning older populations. The limited studies available present mixed results (Adamson et al., 2009; Andersen et al., 2019; Steffens et al., 2024), and the latest research even suggests no major gender differences in overall treatment outcomes (Holzhauer et al., 2020; Steffens et al., 2024). There is a clear need for additional research exploring gender differences in treatment outcomes among older adults. Our findings are also in line with an earlier study from this cohort, which showed significant increases in various quality of life measures over time points, but with no differences between females and males (Tryggedsson et al., 2023). Both studies contribute to a growing body of literature suggesting that gender may not be as significant a predictor of treatment outcomes in older adults with AUD as previously thought. Furthermore, both studies reported consistent improvements in treatment outcomes, regardless of gender, which supports the notion that both older men and women benefit from AUD treatment. These findings highlight that gender, while not always a primary determinant of treatment outcomes, may still influence how drinking behaviors develop during and after treatment. Variations in alcohol metabolism and life circumstances, such as social support, living conditions, and financial or health‐related factors, could also explain the differences in treatment response between males and females observed in other studies (Adamson et al., 2009; Satre et al., 2007; Satre, Mertens, Areán, et al., 2004; Satre, Mertens, & Weisner, 2004).

### Limitations and strengths

This study has potential limitations. We adjusted for a categorized ADS score, acknowledging the limitation that the overall ADS may not align closely with DSM‐5 AUD severity in older adults (Mejldal et al., 2020). Despite the comprehensive approach, the binary nature of certain outcomes (like abstinence and heavy drinking) might oversimplify the complex nature of recovery in AUD, potentially masking subtle but clinically significant effects. Another methodological challenge is the use of BAC as an outcome measure. Research has consistently shown that women experience greater impairment than men after consuming the same amount of alcohol, resulting in a higher BAC, even after adjusting for body weight (Jones & Jones, 1976; Lieber, 1997; Thomasson, 2002). Generally, BAC is influenced by physiological factors that differ between genders, such as body weight and gender‐specific factor multiplication. These factors can lead to discrepancies in BAC between males and females, potentially distorting the perceived effectiveness of treatment interventions. Moreover, no information on ethnicity was included, so potential ethnic differences, including interaction with gender, could not be elucidated. Finally, we were unable to assess whether exclusion due to severe depression differed by gender, as these data were not recorded in the parent trial. This could have influenced the gender distribution in the included sample and may have attenuated observed gender differences in treatment outcomes.

This study also has several notable strengths. Our study utilized a large, multinational dataset, enhancing the generalizability of the findings. A major strength is the use of advanced statistical methods to adjust for potential confounders, handle variability in treatment outcomes, and the comprehensive handling of missing data through multiple imputation. We also compared our results using multiple imputation with results using nonimputed data. Results using the two different approaches were essentially the same. The same applies for the attrition analyses where gender in no way was related to missingness. Results using nonimputed data and attrition analysis results are available upon request. Simply ignoring or choosing the wrong methodological approach to handle missing data, especially in longitudinal studies, can introduce biases whose magnitude and direction are uncertain (Biering et al., 2015). The selection of outcome measures was comprehensive, incorporating a variety of drinking‐related outcomes, both continuous and binary, allowing for a nuanced analysis of gender‐specific treatment efficacy. By investigating gender differences in treatment outcomes using different outcome definitions, this study addresses a gap in the literature.

### Future research

The gender differences and similarities in treatment outcomes observed in older adults may be influenced by various factors, including biological variations in alcohol metabolism, gender‐specific social roles and pressures, and differing comorbidity patterns with other mental health disorders. There is a need for further exploration of how these factors influence treatment outcomes. Future research should explore these underlying mechanisms in more detail, potentially through comprehensive approaches that combine biological assessments, detailed psychosocial evaluations, and extended longitudinal studies. Understanding the underlying factors contributing to these differences could inform the development of more targeted interventions. Studies should also aim at following individuals beyond 1 year after treatment to assess the long‐term stability of treatment effects and to identify potential late‐emerging differences between genders. Additionally, research should focus on how gender differences interact with other individual and demographic factors, such as motivation for change, comorbidities, and life circumstances, to create more personalized treatment strategies for older adults with AUD. Studies that investigate such factors in the development and maintenance of AUD in older adults should include gender as a potentially moderating factor. Our study was not designed to evaluate gender‐specific treatment adaptations, but future research should explore whether tailored approaches—particularly those addressing the unique needs of older women—may enhance treatment efficacy. Finally, while this study focused on 30‐day drinking outcomes at each follow‐up, future research could leverage the full Timeline Follow‐Back (TLFB) data from Form 90 to explore more detailed drinking trajectories over time. Such analyses may uncover gender‐specific patterns not captured by point estimates and further inform personalized approaches to AUD treatment in older adults.

## CONCLUSIONS

In conclusion, this study provides important insights into the gender‐specific effects of AUD treatment outcomes in older adults, demonstrating that the impact of treatment can vary depending on the outcome measures used. While gender‐specific differences in drinking behaviors were evident, with particularly less favorable reductions among women, the adjusted drinking outcomes were broadly comparable across genders, and improvements were sustained up to 1 year after treatment. However, given women's greater physiological vulnerability to alcohol, this may not reflect equivalent clinical risk. Nevertheless, these findings emphasize that, regardless of gender, older adults can achieve meaningful and lasting benefits from AUD treatment.

## FUNDING INFORMATION

This study was unconditionally funded by the Lundbeck Foundation (grant no.: R155‐2012‐11282), the University of Southern Denmark, and the Psychiatric Research Foundation, Region of Southern Denmark.

## CONFLICT OF INTEREST STATEMENT

Nothing to disclose.

## Supporting information


Table S1.

## Data Availability

The data that support the findings of this study are available on request from the corresponding author. The data are not publicly available due to privacy or ethical restrictions.
