# Supplementary material for: Exploring the role of gender on treatment outcomes in older adults with alcohol use disorder
Source: Alcohol Clin Exp Res (Hoboken). 2025 Sep 10;49(11):2553–66. doi: 10.1111/acer.70164 (PMC12638275; doi:10.1111/acer.70164)
Supplement: Supplementary file 1 — Table S1. [file ACER-49-2553-s001.docx]

**Table S1.** Mean values of continuous drinking outcomes over time, stratified by gender, after multiple imputation.

|  | Time | | | | | | | | | |
| --- | --- | --- | --- | --- | --- | --- | --- | --- | --- | --- |
|  | Baseline | | 4 weeks | | 12 weeks | | 26 weeks | | 52 weeks | |
| Gender | Mean | SD | Mean | SD | Mean | SD | Mean | SD | Mean | SD |
| Male |  |  |  |  |  |  |  |  |  |  |
| Drinks per day | 6.35 | 5.49 | 3.06 | 3.76 | 2.92 | 4.07 | 2.84 | 3.54 | 3.15 | 3.93 |
| Drinks per drinking day | 9.39 | 6.95 | 5.17 | 5.31 | 4.67 | 5.17 | 4.72 | 5.32 | 4.59 | 4.82 |
| Percent days abstinent | 36.19 | 35.83 | 55.49 | 40.14 | 59.34 | 40.15 | 57.81 | 37.83 | 52.9 | 41.82 |
| Percent heavy drinking days | 50.29 | 38.22 | 26.94 | 36.32 | 23.58 | 34.51 | 25.27 | 35.73 | 28.08 | 39.13 |
| Female |  |  |  |  |  |  |  |  |  |  |
| Drinks per day | 4.03 | 3.88 | 1.94 | 2.59 | 1.97 | 2.58 | 2.2 | 3.09 | 2.21 | 3.24 |
| Drinks per drinking day | 6.26 | 4.92 | 3.33 | 3.44 | 3.51 | 3.71 | 3.71 | 4.19 | 3.65 | 4.03 |
| Percent days abstinent | 44.53 | 37.73 | 59.48 | 38.48 | 60.73 | 38.26 | 58.4 | 40.21 | 56.67 | 41.59 |
| Percent heavy drinking days | 42.65 | 37.41 | 20.27 | 32.17 | 19.07 | 31.86 | 24.1 | 37.59 | 22.86 | 41.12 |
| Total |  |  |  |  |  |  |  |  |  |  |
| Drinks per day | 5.39 | 5.02 | 2.56 | 3.33 | 2.48 | 3.5 | 2.57 | 3.37 | 2.76 | 3.69 |
| Drinks per drinking day | 8.09 | 6.38 | 4.35 | 4.65 | 4.14 | 4.59 | 4.29 | 4.9 | 4.2 | 4.54 |
| Percent days abstinent | 39.64 | 36.83 | 57.28 | 39.43 | 59.98 | 39.28 | 58.06 | 38.84 | 54.46 | 41.76 |
| Percent heavy drinking days | 47.13 | 38.05 | 23.94 | 34.66 | 21.5 | 33.38 | 24.78 | 36.52 | 25.91 | 40.04 |

Values are estimated using multiple imputation to account for missing data.

Abbreviations: SD, standard deviation.
